# Supplementary material for: Breast cancer metastasis suppressor OTUD1 deubiquitinates SMAD7
Source: Nat Commun. 2017 Dec 13;8:2116. doi: 10.1038/s41467-017-02029-7 (PMC5727433; doi:10.1038/s41467-017-02029-7)
Supplement: Supplementary file 1 — Description of Additional Supplementary Files [file 41467_2017_2029_MOESM1_ESM.docx]

**Description of Additional Supplementary Files**

File Name: Supplementary Data 1

Description: DUB shRNA list used in this study; Related to Figure 1. Supplementary Data 1 shows a list of TRC number of sigma mission shRNA library targeting 74 DUBs, in which each DUB is covered by 4-6 independent short hairpins with at least two of them validated.

File Name: Supplementary Data 2

Description: BRCA1 core enrichment in OTUD1-high patients; Related to Figure 1. We compare NKI295 tumor microarray data 54 OTUD1-high expressing patients and 64 OTUD1-low expressing patients and apply it to Gene Set Enrichment Analysis (GSEA). We observe that about 70 core targets of tumor suppressor Breast Cancer 1 (BRCA1) are all significantly enriched in OTUD1-high patients. Details are listed in Supplementary Data 2.

File Name: Supplementary Data 3

Description: Metastasis-related genes changed upon altered expression of OTUD1; Related to Figure 3, Figure 8 and Supplementary Figure 3. Supplementary Data 3 shows: (1) PCR array analyses in which the loss of OTUD1 lead to several molecular features of mesenchymal cells, including the upregulation of key transcriptional inducers and other EMT-related targets. (2) TGF-β target genes are suppressed by an increase of OTUD1 wt, but not by OTUD1 CA, either in the basal or TGF-β-induced level. (3) TGF-β and HRAS collaborate to promote EMT, invasion and cancer stem traits in breast cancer cells which are enhanced upon depletion of OTUD1 in MCF10A cells.

File Name: Supplementary Data 4

Description: Mass spec analysis identified K220 residue as SMAD7 ubiquitination site; Related to Figure 6. In mass spectrometry analysis, multiple peptides listed identify SMAD7 Lysine 220 to be ubiquitinated.

File Name: Supplementary Data 5

Description: Primer list; Related to Figure 2, Figure 3, Figure 8, Supplementary Figure 3 and Supplementary Figure 6.
