# Supplementary material for: Breast cancer metastasis suppressor OTUD1 deubiquitinates SMAD7
Source: Nat Commun. 2017 Dec 13;8:2116. doi: 10.1038/s41467-017-02029-7 (PMC5727433; doi:10.1038/s41467-017-02029-7)
Supplement: Supplementary file 2 — Supplementary Information [file 41467_2017_2029_MOESM2_ESM.pdf]

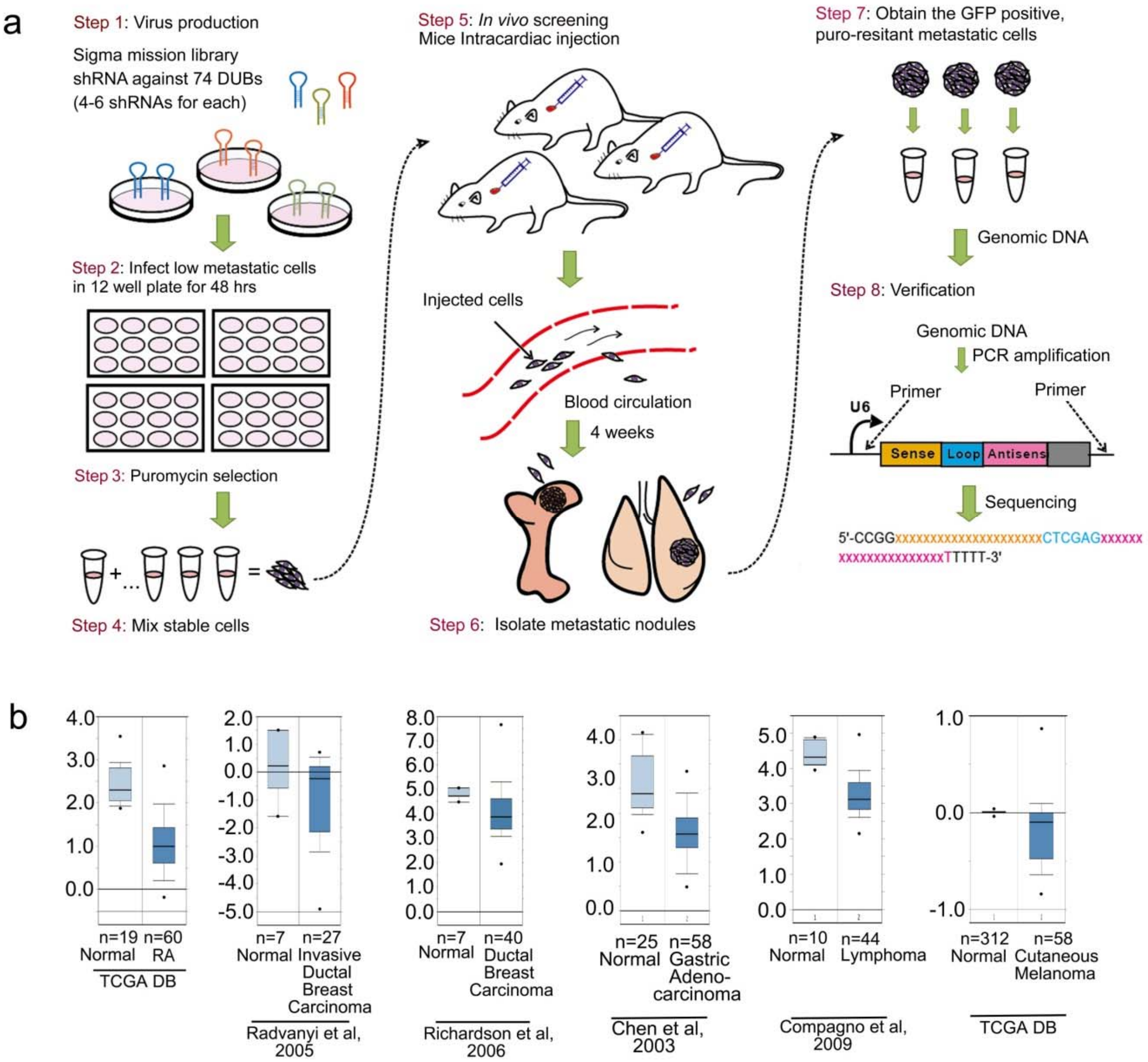

Supplementary Figure 1. Loss of function genetic screen identifies OTUD1 as a metastasis-repressor. (**a**) Schematic representation of an *in vivo* genetic screen for selective DUBs that upon knockdown increase breast cancer cell metastasis. A Sigma mission library of lentiviral shRNA constructs against 74 DUBs (4-6 shRNAs per gene, totally 371 shRNA constructs) was used. Each shRNA was separately produced in HEK293T cells (Step 1) and then individually infected into low metastatic GFP and Luciferase labeled breast cancer MDA-MB-231 cells (Step 2). Cells were selected with puromycin (Step 3) and then mixed (for each shRNA expressing cell line 10 x 10^3 cells were used) (Step 4) for intracardial injection in 30 female, 6 weeks old nude mice ($10x10^4$ cells per mice) (Step 5). Four weeks later, strong metastatic nodules were isolated from injected mice. None of the control shRNA infected MDA-MB-231

cells developed metastasis in this 4 week time period. From the isolated metastatic outgrowths GFP positive/puromycin-resistant cells were selected through puromycin for genomic DNA isolation (Step 7). Sequence of shRNA was amplified and verified by DNA sequencing using U6 primer (Step 8). (**b**) Oncomine box plots of OTUD1 expression levels in multiple human advanced cancers.

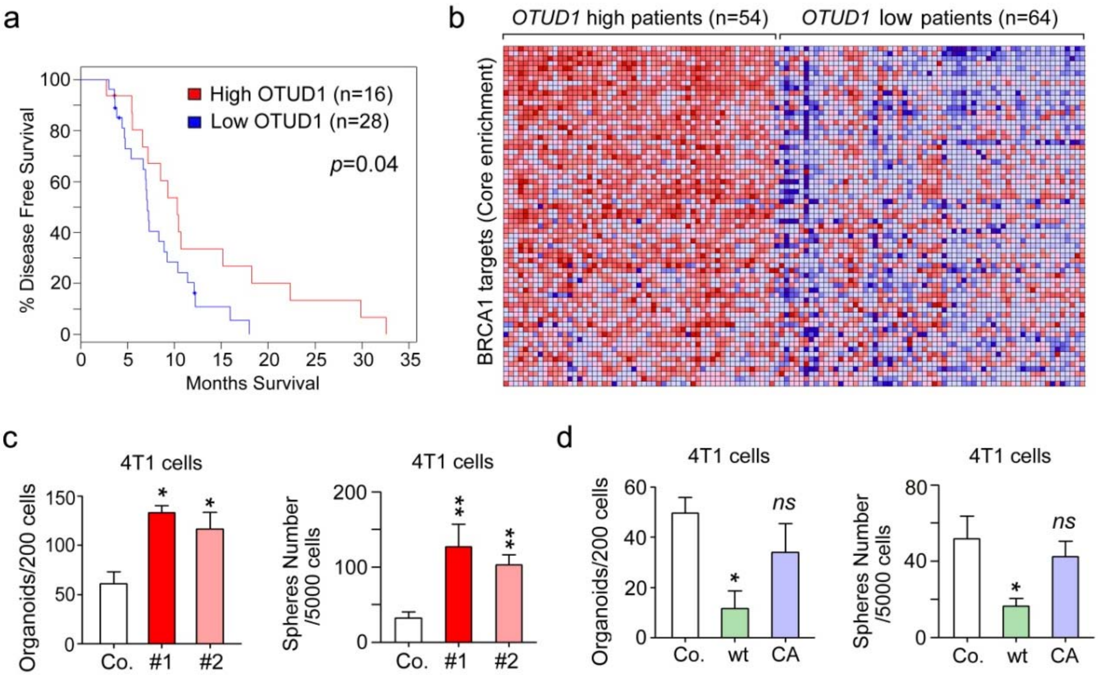

Supplementary Figure 2. ***OTUD1*** **is a tumor suppressor of which the gene product inhibits cancer stem cell traits and enriches BRCA1 core target genes in human breast cancer patients.** (**a**) Kaplan-Meier curves showing that metastasis-free survival of individuals was positively correlated with *OTUD1* expression in bladder urothelial cancer in TCGA database (http://cancergenome.nih.gov/) by log- rank test. (**b**) Pre-ranked gene-set enrichment analysis (GSEA) showing that BRCA1 target genes are upregulated in *OTUD1*-high versus *OTUD1*-low expressing patients. (**c**) Mean number of organoids (±SE) per 200 cells from triplicate samples of control and OTUD1-silenced 4T1 cells cultured in 3D Matrigel (Left). Mean number of tumor spheres per $5x10^3$ cells from triplicate samples of control and OTUD1 silenced 4T1 cells (Right). (**d**) Mean number of organoids (±SE) per 200 cells from triplicate samples of control and OTUD1 wt or CA overexpressing 4T1 cells cultured in 3D Matrigel (Left). Mean number of tumor sphere per $5x10^3$ cells from triplicate samples of control and OTUD1 wt or CA expressing 4T1 cells (Right). For c and d, Error bar, ±SE.P values; Student's t test.

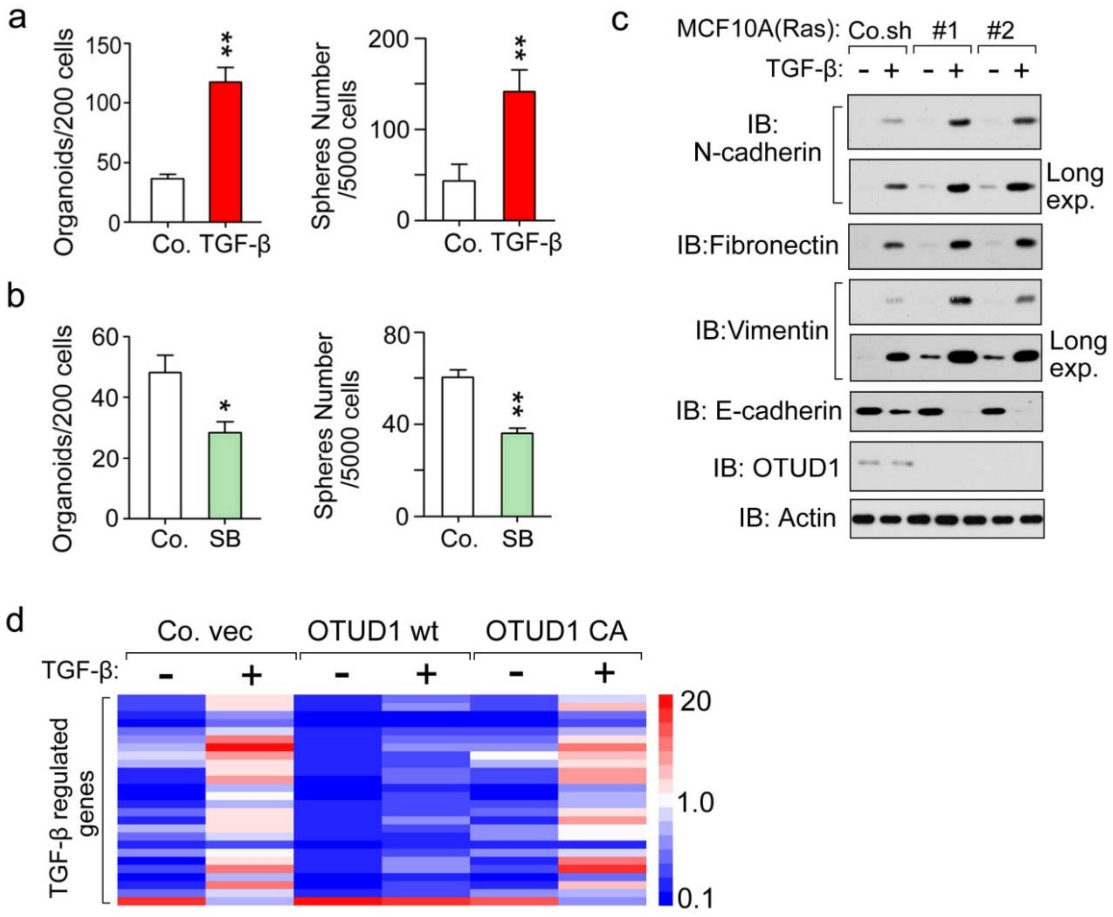

Supplementary Figure 3. OTUD1 inhibits TGF-β-mediated EMT. (**a**) Mean number of organoids (±SE) per 200 cells from triplicate samples of control and TGF-β (5 ng ml^{-1} for 4 days) treated breast cancer MCF10A-RAS cells cultured in 3D Matrigel (Left). Mean number of tumor spheres per 5x10^3 cells from triplicate samples of control and TGF-β (5 ng ml^{-1} for 4 days) treated MCF10A-RAS cells (Right). (**b**) Mean number of organoids (±SE) per 200 cells from triplicate samples of control and TβRI kinase inhibitor SB431542 (10 μM for 4 days) treated MCF10A-RAS cells cultured in 3D Matrigel (Left). Mean number of tumor spheres per 5x10^3 cells from triplicate samples of control and SB431542 (10 μM for 4 days) treated MCF10A-RAS cells (Right). (**c**) Immunoblot (IB) analysis of epithelial (E-Cadherin) and mesenchymal (N-cadherin, Fibronectin and Vimentin) marker protein expression in control (Co.sh) and OTUD1-silenced MCF10A-RAS cells (#1 and #2) treated with TGF-β (2.5 ng/ml) for 72 h. Actin was analysed to control for

equal loading.(**d**) Heat map of qRT-PCR analysis of control (Con.vec) and OTUD1 wt/CA-overexpressing MCF10A-RAS cells treated with or without TGF-β (2.5 ng/ml) for 8 h. For details see Supplementary Table S4.

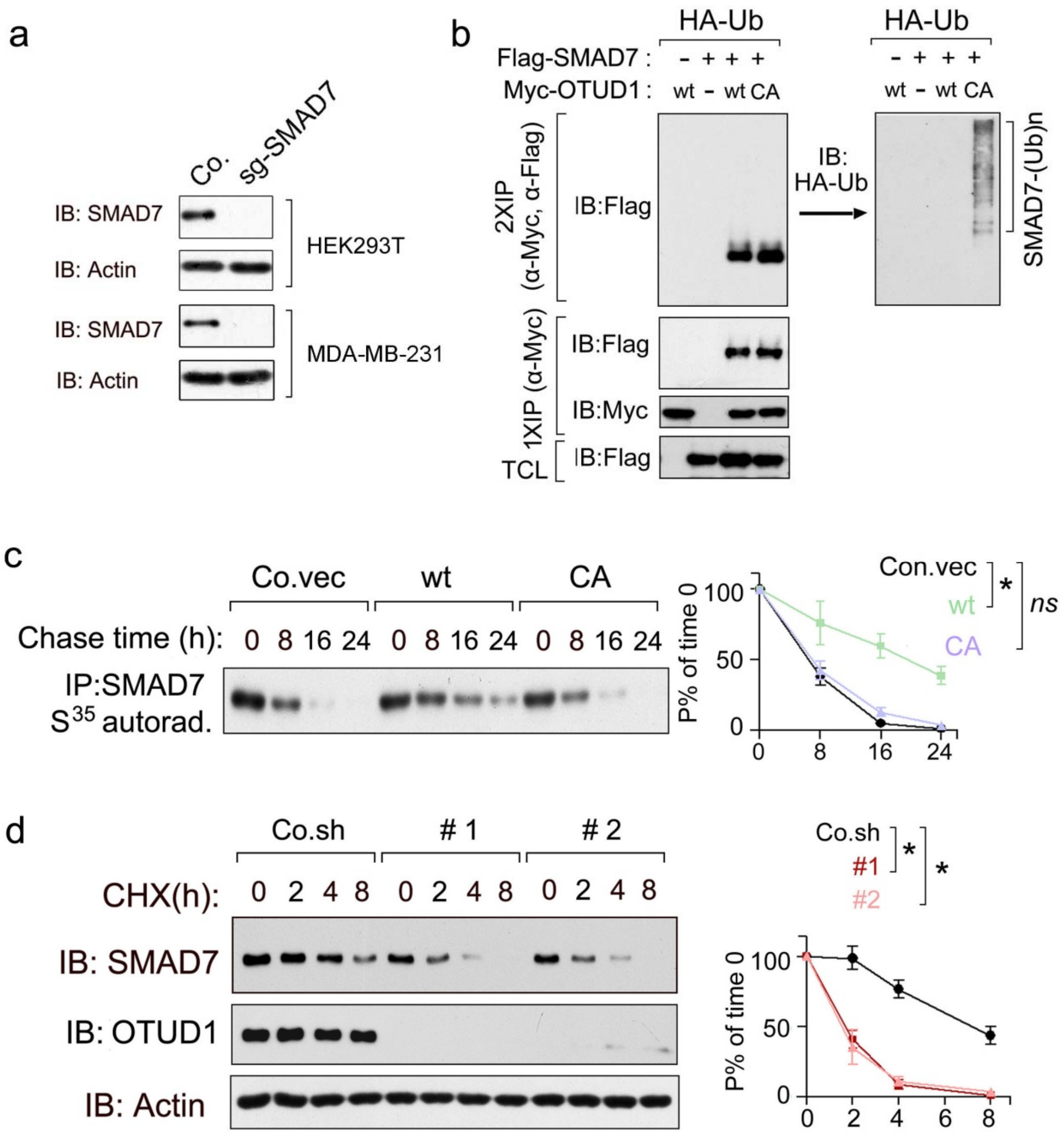

Supplementary Figure 4. OTUD1 deubiquitylates SMAD7 and controls SMAD7 stability. (**a**) Immunoblot (IB) analysis of SMAD7 sgRNA mediated knock out in HEK293T and the early passage breast cancer MDA-MB-231 cells. Actin was analysed to control for equal loading. (**b**) Stable HA-Ub expressing HEK293T cells were transfected with Flag-SMAD7 and Myc-OTUD1 wt or CA as indicated. Cells were treated with MG132 (5 μM) for 4h before harvest. 48 h upon transfection, cells were harvested for a (first) immunoprecipitation with anti-Myc antibody and then eluted with 2% SDS and thereafter 20 fold diluted (resulting in a 0.1% SDS concentration), followed by a (second) immunoprecipitation with anti-Flag M2 beads.

Total cell lysate and sequential immunoprecipitates were analyzed by IB. (**c**) [^{35}S]-methionine labeling and pulse-chase studies of SMAD7 in control (Co.vec) and OTUD1-overexpressing wt/CA cells. The amount of precipitated labeled protein after the chase was expressed as the percentage of that at the beginning of the chase (time 0) and shown in the right panel. Results are shown as means ± SD of two independent sets of experiments in duplicate. (**d**) IB of lysates derived from control (Co.sh) and stable OTUD1- depleted HEK293T cells (#1 and #2) treated with cycloheximide (CHX, 20 μg/ml) at indicated time points. Actin was loaded as an internal control. Quantification of the band intensities is shown in the right panel. Band intensity was normalized to the t=0 controls. Results are shown as means ± SD of three independent sets of experiments.

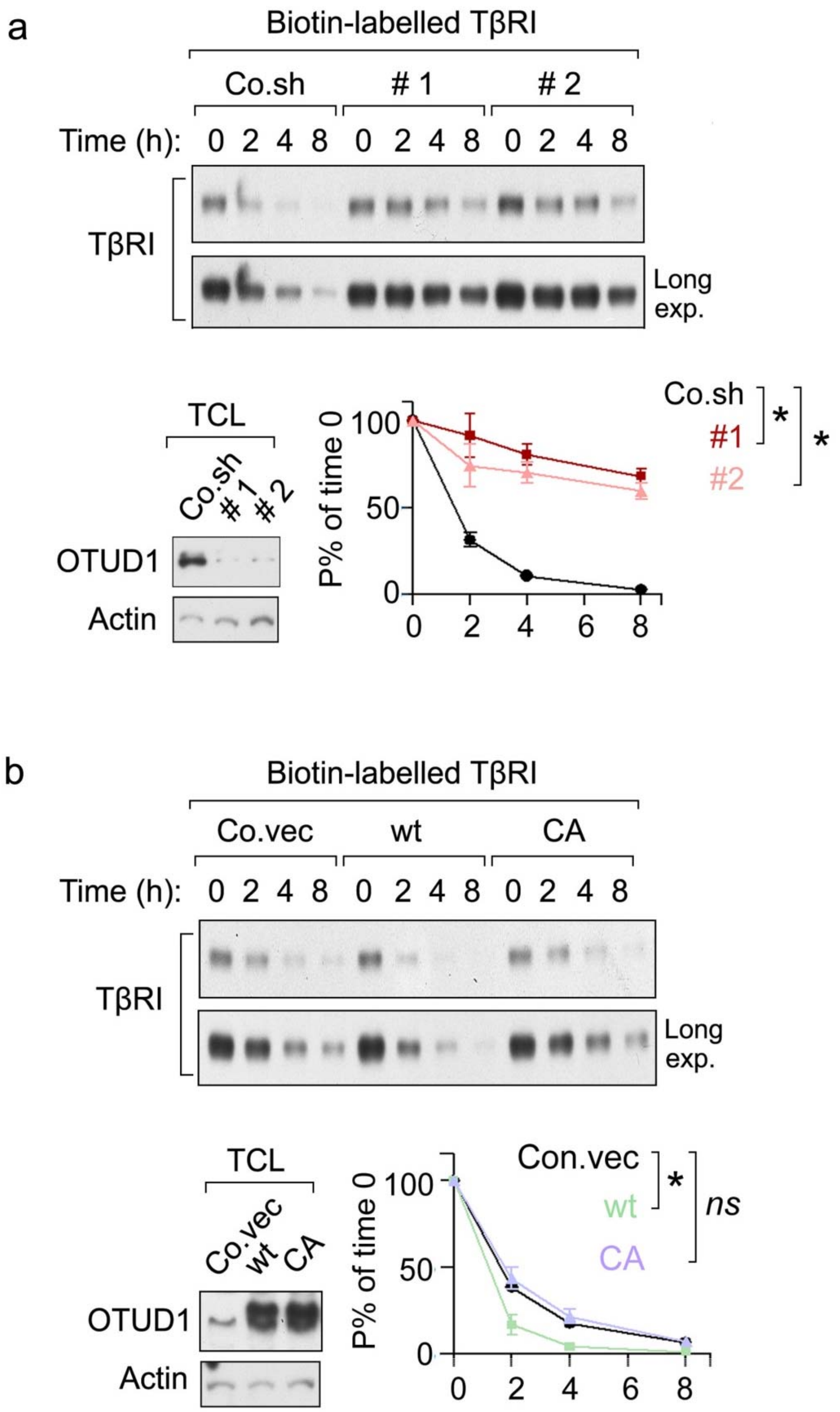

Supplementary Figure 5. OTUD1 promotes the turnover of cell surface TβRI. (**a**) Immunoblot (IB) of biotinylated cell surface TβRI in control (Co.sh) and stable OTUD1-depleted HeLa cells (#1 and #2) treated with TGF-β (5 ng ml^{-1}) for the indicated time points. OTUD1 knockdown efficiency is shown in the lower left panel. Quantification of the band intensities is shown in the lower right panel. Band intensity

was normalized to the t=0 controls. Results are shown as means ± SD of three independent sets of experiments. (**b**) IB of biotinylated cell surface TβRI in HeLa stably expressing empty vector (Co.vec),OTUD1 wt or CA and treated with TGF-β (5 ng ml^{-1}) for the indicated time points. Expression of OTUD1 wt or CA is shown in the lower left panel. Quantification of the band intensities is shown in the lower right panel. Band intensity was normalized to the t=0 controls. Results are shown as means ± SD of three independent sets of experiments.

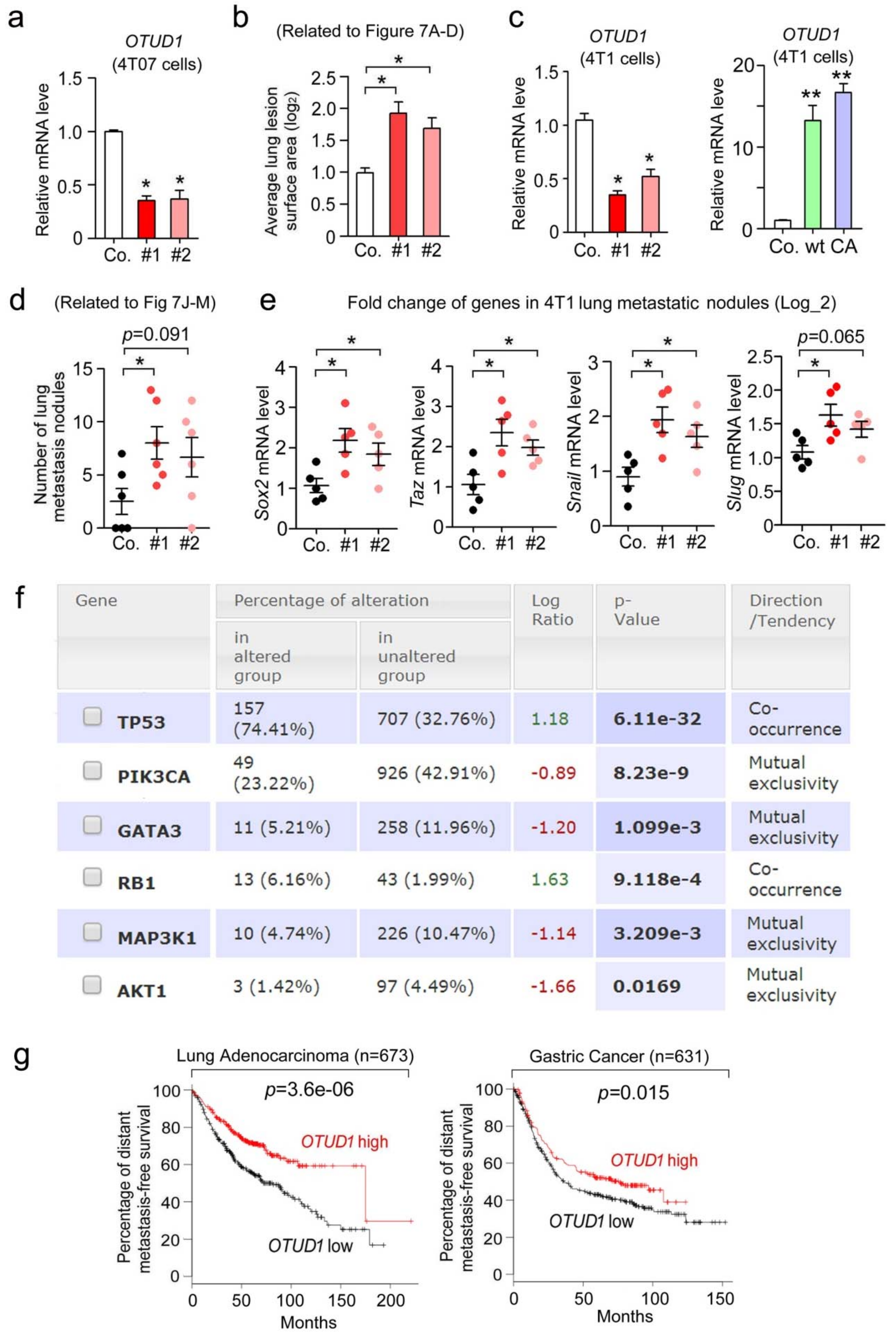

Gene	Percentage of alteration in altered group	Percentage of alteration in unaltered group	Log Ratio	p-Value	Direction /Tendency
TP53	157 (74.41%)	707 (32.76%)	1.18	6.11e-32	Co-occurrence
PIK3CA	49 (23.22%)	926 (42.91%)	-0.89	8.23e-9	Mutual exclusivity
GATA3	11 (5.21%)	258 (11.96%)	-1.20	1.099e-3	Mutual exclusivity
RB1	13 (6.16%)	43 (1.99%)	1.63	9.118e-4	Co-occurrence
MAP3K1	10 (4.74%)	226 (10.47%)	-1.14	3.209e-3	Mutual exclusivity
AKT1	3 (1.42%)	97 (4.49%)	-1.66	0.0169	Mutual exclusivity

Supplementary Figure 6. OTUD1 inhibits lung metastasis in transplantable mouse models and correlates with good prognosis in cancers. (a) QRT-PCR

analysis of shRNA-mediated *OTUD1* mRNA knock down efficiency in breast cancer 4T07 cells. (**b**) Related to Fig. 7a-d, control (Co.) or OTUD1-silenced (sh#1 and sh#2) 4T07 cells were tail vein-injected into syngeneic BALB/c mice. Average lung lesion surface area (±SD) (arbitrary units based on pixel quantification from digital images) are shown. (**c**) QRT-PCR analysis of *OTUD1* knock down efficiency or *OTUD1* ectopic expression in 4T1 cells. (**d**) Control (Co.) or OTUD1-depleted 4T1 cells were orthotopic injected in syngeneic BALB/c mice. Lung metastasis nodules in each group were calculated (±SD). (**e**) QRT-PCR analysis of *Sox2, Taz, Snail, Slug* mRNA levels in 4T1 lung-metastatic nodules; n=5 per group. (**f**) Related to Figure 8B, the rare breast cancer patients with gain or amplification of OTUD1 have co-occurrence with p53 expression and mutual exclusivity with expression of PIK3CA and AKT1. (**g**) Kaplan-Meier curves showing that metastasis-free survival of individuals is positively correlated with *OTUD1* expression in lung adenocarcinoma (left panel) and gastric cancer (right panel).

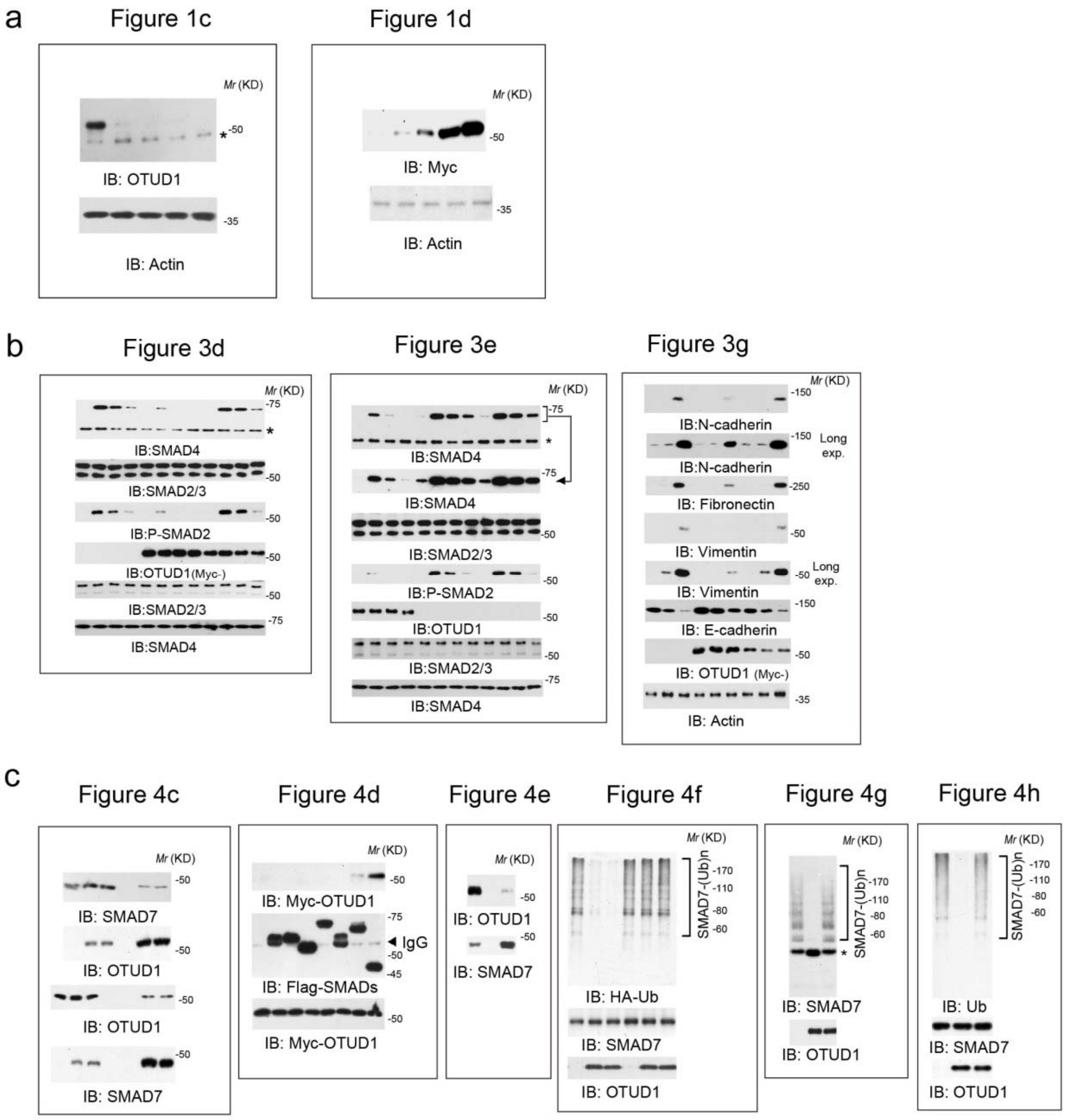

Supplementary Figure 7. Uncropped and unmodified data related to Figure 1, Figure 3 and Figure 4. (**a**) Data for Figure 1c and Figure 1d. (**b**) Data for Figure 3d, Figure 3e and Figure 3g. (**c**) Data for Figure 4c, Figure 4d, Figure 4e, Figure 4f, Figure 4g and Figure 4h.

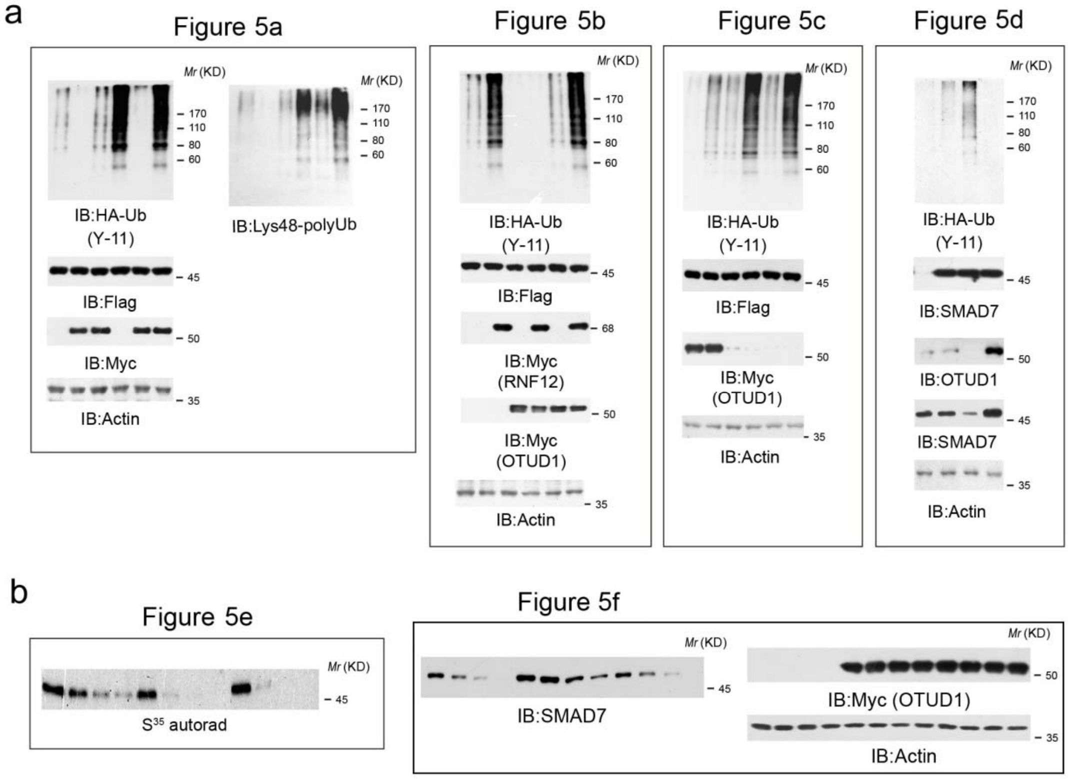

Supplementary Figure 8. Uncropped and unmodified data related to Figure 5. (**a**) Data for Figure 5a, Figure 5b, Figure 5c and Figure 5d. (**b**) Data for Figure 5e and Figure 5f.

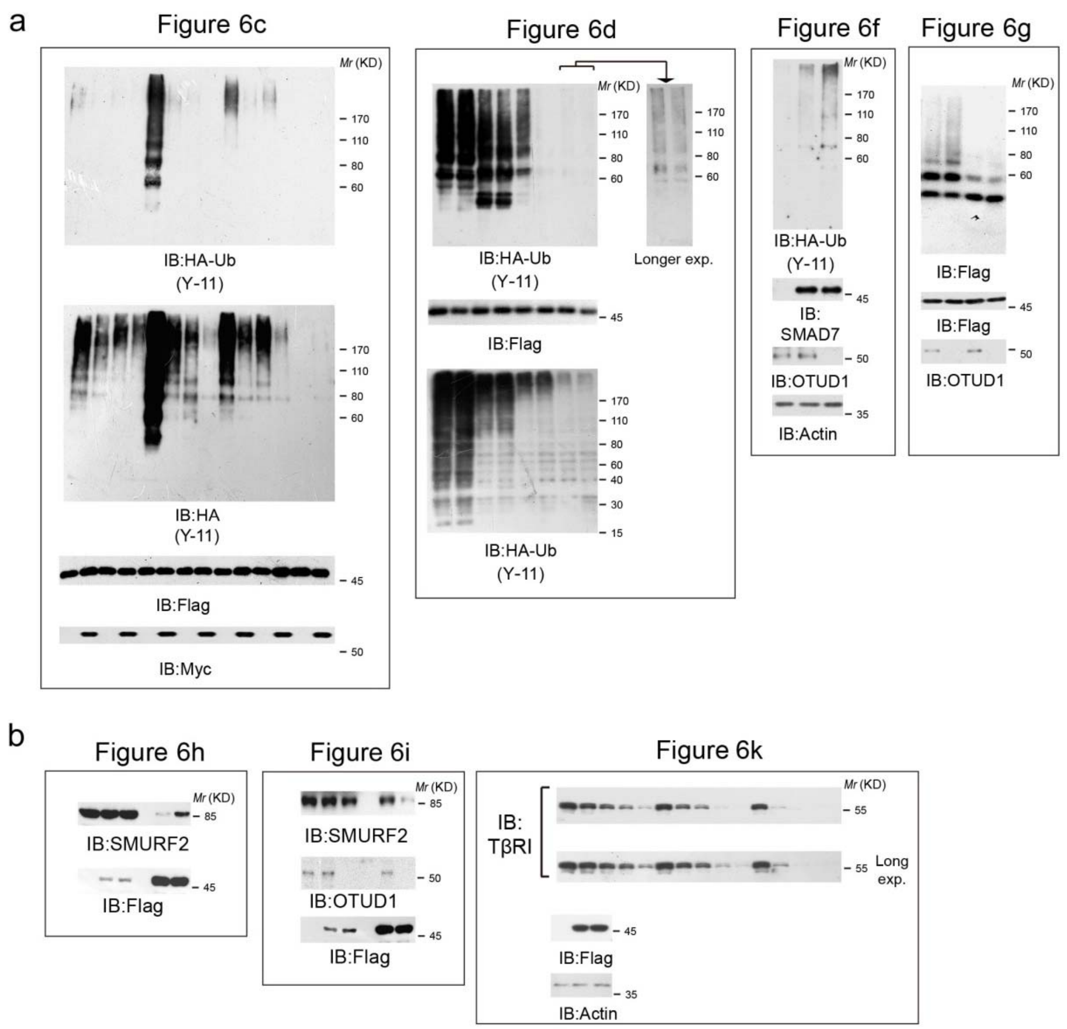

Supplementary Figure 9. Uncropped and unmodified data related to Figure 6. (**a**) Data for Figure 6c, Figure 6d, Figure 6f and Figure 6g. (**b**) Data for Figure 6h, Figure 6i and Figure 6k.

Supplementary Table 1 Tumor initiating frequency

Primary Tumor-initiating Capacity			
MCF10A (Ras) cells	Number of Cells	Tumor incidence	Tumor iniciating Frequency
Control sh	100	6 of 10	<1/50
	1000	9 of 10	
	10000	10 of 10	
	100000	10 of 10	
sh-OTUD1 #1	100	10 of 10	1
	1000	10 of 10	
	10000	10 of 10	
	100000	10 of 10	
sh-OTUD1 #2	100	9 of 10	0.9
	1000	10 of 10	
	10000	10 of 10	
	100000	10 of 10	

Primary Tumor-initiating Capacity			
MCF10A (Ras) cells	Number of Cells	Tumor incidence	Tumor iniciating Frequency
Control vec	100	10 of 10	1
	1000	10 of 10	
	10000	10 of 10	
	100000	10 of 10	
OTUD1 wt	100	5 of 10	<1/1000
	1000	8 of 10	
	10000	10 of 10	
	100000	10 of 10	
OTUD1 CA	100	9 of 10	0.9
	1000	10 of 10	
	10000	10 of 10	
	100000	10 of 10	
Tumor-initiating Capacity was calculated in http://bioinf.wehi.edu.au/software/elda/			

Supplementary Table 2 Deletion and Loss of OTUD1 gene copies

		n	Deletion (%)	Loss (%)
1	GBM(TCGA)	273		88.3
2	chRCC(TCGA)	66		72.7
3	Melanoma(TCGA)	287	0.7	51.6
4	Testicular germ cell(TCGA)	149	0.7	51
5	Sarcoma(TCGA)	243	0.4	45.3
6	Lung squ(TCGA)	177	1.7	42.4
7	Pancreas(UTSW)	109	0.9	43.1
8	LGG-GBM(TCGA 2016)	794		42.6
9	Protate(FHCRC,2016)	136	1.5	37.5
10	CCLE(Novartis/Broad 2012)	881	2.5	33.8
11	Head & neck(TCGA)	504	1.2	31.3
12	Uterine CS(TCGA)	56		32.1
13	NSCLC(TCGA 2016)	1144		30.2
14	Esophagus(TCGA)	184	0.5	26.1
15	Prostate(MICH)	61		26.2
16	Cervical(TCGA)	191		22
17	Bladder(TCGA)	127	1.6	17.3
18	Colorectal(TCGA)	220		16.4
19	Glioma(TCGA)	283	0.4	15.2
20	Stomach(TCGA)	393		14.8
21	Breast(TCGA)	963		13.2
22	Ovarian(TCGA)	311		12.2
23	Breast(METABRIC)	2051		8
